# Oral administration of a select mixture of *Bacillus* probiotics generates Tr1 cells in weaned F4ab/acR^−^ pigs challenged with an F4^+^ ETEC/VTEC/EPEC strain

**DOI:** 10.1186/s13567-015-0223-y

**Published:** 2015-09-17

**Authors:** Dong Zhou, Yao-Hong Zhu, Wei Zhang, Meng-Ling Wang, Wen-Yi Fan, Dan Song, Gui-Yan Yang, Bent Borg Jensen, Jiu-Feng Wang

**Affiliations:** College of Veterinary Medicine, China Agricultural University, Beijing, 100193 China; Division of Microbiology and Immunology, Danish Center for Food and Agriculture, Aarhus University, DK 8830 Tjele, Denmark

## Abstract

Although breeding of F4 receptor − negative (F4R^−^) pigs may prevent post-weaning diarrhea, the underlying immunity is poorly understood. Here, various doses of a *Bacillus licheniformis* and *Bacillus subtilis* mixture (BLS-mix) were orally administered to F4ab/acR^−^ pigs for 1 week before F4 (K88) − positive ETEC/VTEC/EPEC challenge. Administration of BLS-mix increased the percentage of Foxp3^−^IL-10^+^ T cells but not of Foxp3^+^IL-10^+^ regulatory T (Treg) cells among peripheral blood CD4^+^ T cells. A low dose of BLS-mix feeding resulted in increased the expression of *IL-6*, *TNF-α*, *IL-10*, and the transcription factors *Foxp3* and *T-bet* mRNAs in the jejunum. Administration of either a low or high dose BLS-mix also led to an increase in the percentage of CD4^+^Foxp3^+^ Treg cells among intraepithelial lymphocytes and CD4^+^IL-10^+^ T cells in the small intestinal Peyer’s patches and the lamina propria of F4ab/acR^−^ pigs following F4^+^ ETEC/VTEC/EPEC challenge. The increased number of IL-10–producing CD4^+^ T cells was attributed to an increase in the proportion of Foxp3^−^IL-10^+^ Treg cells rather than Foxp3^+^IL-10^+^ Treg cells. Our data indicate that oral administration of BLS-mix to newly weaned F4ab/acR^−^ pigs ameliorates enteritis in an F4^+^ ETEC/VTEC/EPEC model; however, induction of IL-10–producing Foxp3^−^ Treg cells by BLS-mix administration cannot account for the protection of newly weaned F4ab/acR^−^ pigs from F4^+^ ETEC/VTEC/EPEC infection, and that excessive generation of CD4^+^IL-10^+^ T cells following consumption of BLS-mix during episodes of intestinal inflammation that is caused by enteric pathogens might prohibit clearance of the pathogen. Select probiotic mixtures may allow for tailoring strategies to prevent infectious diseases.

**Electronic supplementary material:**

The online version of this article (doi:10.1186/s13567-015-0223-y) contains supplementary material, which is available to authorized users.

## Introduction

Enterotoxigenic *Escherichia coli* (ETEC) harboring F4 (K88)^+^ fimbriae are a common cause of diarrhea in neonatal and newly weaned pigs [1]. Adhesion of ETEC to the intestinal epithelium is a very complex process that may involve several structures including fimbriae and flagella [2]. Attachment of F4 fimbriae to specific F4 receptors on the porcine intestinal brush border is the first step in the infection process. Following colonization of the gut, F4^+^ ETEC secrete heat-labile (LT) and/or heat-stable (ST) enterotoxins or release lipopolysaccharide (LPS), which lead to diarrhea, intestinal inflammation and/or fever. In addition, ETEC and other diarrhoeagenic *E. coli* (e.g. verocytotoxigenic *E. coli* [VTEC], enteropathogenic *E. coli* [EPEC]) are able to acquire virulence factors via horizontal gene transfer leading to the development of enteric diseases [3-5].

F4 fimbriae present 3 different antigenic variants (F4ab, F4ac and F4ad), of which F4ac is the most common. Although the exact mutation causing susceptibility to F4^+^ ETEC remains unknown, a polymorphism within an intron of the mucin 4 (*MUC4*) gene on porcine chromosome 13 has been proposed as a candidate gene for the production of the specific ETEC F4ab/ac receptor, and a DNA marker − based test has been developed to allow genotyping for determining ETEC F4ab/ac resistance/susceptibility [6-8]. The locus for the intestinal ETEC F4ac receptor (F4acR) has been mapped to pig chromosome 13q41 with known homology to human chromosome 3q21 and q29, and MUC4 had the most significant linkage with the F4acR locus [9-12]. Thus, breeding of F4 receptor − negative (F4R^−^) pigs is an alternative strategy for preventing F4^+^ ETEC-associated post-weaning diarrhea. However, the immunity of F4R^−^ pigs must be considered in breeding for F4^+^ ETEC resistance, as heterozygous F4R^−^ piglets are not passively protected from infection by these strains.

Active intestinal mucosal immunization is needed for the protection of newly weaned pigs because they have been deprived of passive lactogenic immunity. Probiotic bacteria can modulate host gastrointestinal immune responses to promote health [13]. Our previous studies using an F4^+^ ETEC model of piglet diarrhea demonstrated that pretreatment with certain probiotics, such as *Lactobacillus rhamnosus*, ameliorates infectious diarrhea in newly weaned piglets; however, there is a risk that high-dose *L. rhamnosus* pretreatment may negate the preventative effects [14-16]*.* Bacterial spores are much more resistant to the listed conditions in the stomach and small intestine than living probiotic microorganisms like lactic acid bacteria. Hence they can reach the intestine in higher concentrations and might be more effective as probiotics [17]. *Bacillus subtilis* and *Bacillus licheniformis* had been used in human and livestock decades for regulation of innate and adaptive immune responses [18-20]. But the exact mechanism of *Bacillus subtilis* and *Bacillus licheniformis* for protecting the host against enteric pathogens is not yet fully understood. It remains to be determined whether the administration of select mixtures of probiotics is more effective than administration of single strains in preventing infectious diseases. Thus, there is a compelling need to discover organisms that elicit more robust therapeutic responses, are compatible with the host, and can affect a specific arm of the host immune system in a well-controlled, physiologic manner.

Regulatory T (Treg) cells are a developmentally and functionally distinct T-cell subpopulation that is engaged in controlling inflammation and maintaining intestinal homeostasis [21-23]. In humans and mice, the major Treg-cell populations in the intestine are CD4^+^Foxp3^+^ Treg cells and T regulatory type 1 (Tr1) cells that produce IL-10 [24]. In the small intestine and Peyer’s patches (PPs), Foxp3^−^IL-10^+^ Treg cells were the most prevalent type and demonstrate a cytokine profile, proliferative response and suppressive function typical of Tr1 cells [25]. A high frequency of Tr1-like cells patrol the intraepithelial layer, whereas both Tr1-like cells and Foxp3^+^ Treg cells populate the lamina propria [24]. In swine, Treg cells, primarily the CD4^+^CD25^dim^ subset, have been shown to produce IL-10 [26].

Enhancing Tr1- or Treg-cell function represents a potential strategy for treating human inflammatory bowel disease [24]. The probiotic *Bifidobacterium breve* has been shown to induce generation of Tr1 cells in the colon [27]. However, the clinical efficacy of single-strain probiotics developed to date has been modest. It has been shown that administration of a select mixture of the probiotics including *L. casei* and *Bifidobacterium bifidium* induces the generation of CD4^+^Foxp3^+^ Treg cells from the CD4^+^CD25^−^ T-cell population in mesenteric lymph nodes in mice [28]. A recent study found that oral administration of a rationally select mixture of *Clostridium* strains from the human microbiota induces the accumulation of CD4^+^Foxp3^+^ Treg cells in the colon lamina propria in germ-free mice [29]. In pigs, administration of *Bacillus cereus* var. toyoi results in an increase in the number of lamina propria CD25^+^ lymphocytes [30]. However, the mechanism underlying the probiotic-induced expansion of IL-10–producing T cells in the intestine of pigs has yet to be elucidated.

In the present study, we examined the effects of a select probiotic mixture comprised of *Bacillus licheniformis* and *Bacillus subtilis* (BLS-mix) on the populations of CD4^+^Foxp3^+^ Treg and Tr1 cells in the small intestine of newly weaned F4ab/acR^−^ pigs following F4^+^ ETEC/VTEC/EPEC challenge.

## Materials and methods

### Ethics statement

All animals were treated in strict accordance with the *Guidelines for Laboratory Animal Use and Care* from the Chinese Center for Disease Control and Prevention and the *Rules for Medical Laboratory Animals* from the Chinese Ministry of Health, under protocol CAU-AEC-2013-073 approved by the Animal Ethics Committee of China Agricultural University, as described previously [15]. All surgeries were performed under sodium pentobarbital anesthesia, and every effort was made to minimize suffering.

### DNA marker − based test for detection of F4 receptor genes

The test for the detection of the F4 receptor genes relied on an *Xba*I polymorphism in intron 7 of the porcine mucin 4 gene, as described previously, with minor modifications [6,31]. Briefly, blood samples were collected from the jugular vein of piglets at 5 days of age. DNA was extracted from 100 μL of EDTA-stabilized blood using a QIAamp DNA Mini Kit (Qiagen Inc., Mississauga, ON, Canada). The PCR product obtained from pig genomic DNA is 367 bp in length, and 10 μL of the PCR product was digested with *Xba*I (TakaRa Biotechnology Inc., Dalian, China), as recommended by the protocol. The resistant allele is not digested by *Xba*I, whereas the susceptible allele is digested into 151- and 216-bp fragments. The PCR-restriction fragment length polymorphism assay permitted discrimination between F4ab/ac receptor − positive (F4ab/acR^+^) and − negative (F4ab/acR^−^) pigs.

### Animals

Piglets were selected on the basis of their F4ab/ac resistance genotype. At 5 days of age, piglets were subjected to a DNA marker − based test with DNA extracted from EDTA-stabilized blood samples.

A total of 32 F4ab/acR^−^ crossbred (Landrace × Large White) piglets of mixed gender, selected from 8 different litters, weaned at 21 days of age, and weighing 6.80 ± 0.44 kg, were obtained from Beijing Hog Raising and Breeding Center. At weaning (day 0), pigs were transported to the animal experimental facility of the College of Veterinary Medicine, China Agricultural University. The animals were individually housed in wire-mesh pens, each of which was equipped with a single feeder and nipple drinker, and the pigs were fed a standard weaning diet containing 22.3% crude protein and 14.0 MJ/kg of dietary energy. Feed and water were provided *ad libitum*. None of the diets contained antibiotics and no drug was administered throughout the trial. Prior to the start of the trial, no clinical signs of diarrhea or other diseases were observed in any of the piglets. The pigs were weighed before the start of the trial (day 0), at day 8 (pre-challenge), and at day 15 (when sacrificed). The feed intake of each pig was recorded.

### Bacterial strains

The probiotic mixture Bioplus® YC was kindly provided by Chr. Hansen A/S (Hørsholm, Denmark). The microbial feed additive is an equal mixture of spray-dried spore-forming *Bacillus licheniformis* (DSM 5749) and *Bacillus subtilis* (DSM 5750) at a minimum concentration of 3.2 × 10^9^ viable spores/g. The probiotic mixture was resuspended in sterile physiological saline. Two different doses of the probiotic mixture were prepared: a low dose containing 3.9 × 10^7^ CFU/mL and a high dose containing 7.8 × 10^7^ CFU/mL.

An *Escherichia coli* F4-producing strain (O149:K91, K88ac), obtained from the China Veterinary Culture Collection Center, was grown in Luria-Bertani medium (Oxoid, Basingstoke, England). After overnight incubation at 37 °C with shaking, the culture was diluted 1:100 with fresh Luria-Bertani medium. Following incubation, bacterial cells were harvested by centrifugation at 3000 × *g* for 10 min at 4 °C, washed with sterile physiological saline and resuspended in sterile physiological saline. An *E. coli* inoculum containing 1.0 × 10^9^ CFU/mL was then prepared.

### Detection of selected virulence factors by real-time PCR assay

Detection of the selected virulence factors STa, STh, STp, STb, LT, Shiga-like toxin 2e (*stx2e*), cytotoxic necrotizing factor 1 (*cnf1*), *cnf2*, as well as the locus of the enterocyte effacement (LEE)-encoded virulence factors the *E. coli* attaching and effacing factor intimin (*eae*), translocated intimin receptor (*Tir*), *escV* and *E. coli*-secreted protein A (*espA*) by real-time PCR assay was previously described [32,33]. Briefly, bacterial cells were recovered from 1 mL of culture of *E. coli* grown with shaking at 180 rpm for 18 h at 37 °C in an orbital shaker. Genomic DNA was extracted by using TIANamp Bacteria DNA Kit (Tiangen Biotech Inc., Beijing, China). The sequences of the primers used are listed in Additional file 1.

### Experimental design

On the day of weaning (day 0), pigs were assigned to 4 groups (*n* = 8 per group) and organized by weight and ancestry. Each group received a different treatment, as follows: (1) control (CONT) group (oral administration of sterile physiological saline); (2) ETEC group (oral administration of sterile physiological saline and oral challenge with *E. coli*); (3) LDBE group (oral administration of low-dose probiotic mixture (3.9 × 10^7^ CFU/mL) and oral challenge with *E. coli*); (4) HDBE group (oral administration of high-dose probiotic mixture (7.8 × 10^7^ CFU/mL) and oral challenge with *E. coli*). At 9 h each day on days 1 through 7, pigs in the CONT and ETEC groups were administered 10 mL of sterile physiological saline orally, whereas pigs in the LDBE and HDBE groups received an equal volume of low-dose (3.9 × 10^7^ CFU/mL, once daily) or high-dose (7.8 × 10^7^ CFU/mL, once daily) probiotic mixture solution, respectively, via oral administration. At 9 h on day 8, pigs in the ETEC, LDBE and HDBE groups were administered 10 mL of *E. coli* culture (10^9^ CFU/mL) orally, whereas pigs in the CONT group received the same volume of sterile physiological saline. The probiotic groups did not received any more probiotics after *E. coli* challenge.

### Clinical examinations

The health of each animal was closely monitored throughout the experiment. Rectal temperature was measured twice daily, before feeding at 07:30 h and 19:30 h. Severity of diarrhea was scored according to previously described criteria [15].

### Blood sampling

Blood samples were collected from the jugular vein immediately prior to *E. coli* challenge (0 h) and at 6, 12, 24, 48, 96, and 144 h after challenge. Blood samples were collected into Venoject glass tubes (Terumo Europe NV, Leuven, Belgium) containing EDTA and analyzed fresh for total and differential blood leukocyte counts. Blood samples without additives were centrifuged and the serum was stored at −20 °C until subsequent analysis. A 5-mL aliquot of each blood sample collected at 0, 24, and 144 h and containing sodium heparin was analyzed by flow cytometry.

### Intestinal tissue sampling

For histopathological analysis, proximal, mid-, and distal segments of the jejunum and ileum were rinsed with PBS immediately after opening and were then segmentally divided and immersed in 4% paraformaldehyde. For mRNA analysis, segments of the jejunum and ileum were collected from each pig and immediately frozen in liquid nitrogen and stored at −80 °C. For flow cytometry analysis, sparse jejunal Peyer’s patches, continuous ileal Peyer’s patches, and proximal and distal jejunum and ileum segments (free of associated PPs) were rinsed with PBS immediately after opening, divided segmentally, and incubated in Ca^2+^- and Mg^2+^-free HBSS (10 mM HEPES, 50 U/mL of penicillin, and 50 μg/mL of streptomycin).

### Histopathological scoring

To assess the small intestine pathology, proximal, mid-, and distal segments (approximately to 10 × 15 × 3 mm) of the jejunum and ileum were fixed in 4% paraformaldehyde and paraffin-embedded tissue sections (3 μm) were then stained with hematoxylin and eosin. Inflammation was scored as follows: epithelial integrity (0 = no change, 1 = shedding of <10 epithelial cells per lesion, 2 = shedding of 11 to 20 epithelial cells per lesion, 3 = epithelial ulceration); central lacteal expansion (0 = no change, 1 = mild, 2 = moderate, and 3 = profound); leukocyte infiltration (0 = <10 leukocytes per field, 1 = 11**-**15 leukocytes per field, 2 = 16**-**20 leukocytes per field, 3 = >20 leukocytes per field); and submucosal edema (0 = no change, 1 = mild, 2 = moderate, and 3 = profound). The summation of the scores for each parameter provides an overall inflammation score for each sample, with a range of 0-12. The typical features associated with each grade of inflammation were as follows: 0, normal; 1 to 5, mild inflammation; 6 to 10, moderate inflammation; and 11 or 12, severe inflammation. The jejunum and ileum were assessed separately, and 3 separate sections from each segment were examined. The scoring was performed in a blinded manner. The total jejunal or ileal score for each animal was then calculated as the average of the individual scores from the sections of proximal, mid-, and distal jejunum or ileum. Images of hematoxylin- and eosin-stained tissues were visualized and photographed using an Olympus BX41 microscope (Olympus, Tokyo, Japan) equipped with a Canon EOS 550D camera head (Canon, Tokyo, Japan).

### Immunofluorescence

Attachment of F4^+^ ETEC/VTEC/EPEC to ileal mucosa was determined by an indirect immunofluorescence assay. Ileal tissue samples fixed with 4% paraformaldehyde were embedded in paraffin, cut into 4-μm sections, and collected on silanized slides. The sections were then incubated with rabbit anti-F4 fimbriae antiserum (China Institute of Veterinary Drug Center, Beijing, China) overnight in a humidified chamber at 4 °C. Goat anti-rabbit Cy3-conjugated (AP307F; Sigma-Aldrich) was used as secondary antibody, and DAPI 3 (Sigma-Aldrich) was used for nuclear staining. The slides were visualized and photographed using a Nikon Eclipse T*i*-U inverted fluorescence microscope equipped with a Nikon DS cooled camera head (Nikon).

### Enzyme-linked immunosorbent assay (ELISA)

Porcine-specific commercially available ELISA kits were used to measure the serum concentrations of TNF-α (R&D Systems, Minneapolis, MN), IL-17A (Bethyl Laboratories, Montgomery, TX), IL-10 (LifeSpan Biosciences, Seattle, WA, USA), and IgA (Bethyl Laboratories).

### Isolation of intestinal mucosal lymphocytes

Intestinal cells were isolated as previously described [16]. Briefly, intraepithelial lymphocytes (IELs), lamina propria lymphocytes (LPLs), and Peyer’s patch lymphocytes (PPLs) were isolated from fragments of proximal and distal jejunum and ileum as well as Peyer’s patches. Lymphocytes were then purified using Percoll gradient centrifugation (GE Healthcare, Piscataway, NJ, USA) and collected from the interface between the 40% and 70% Percoll layers.

Suspensions of tissue cells from each intestinal compartment were adjusted to a density of 1 × 10^6^ cells/mL with 1× PBS (containing 0.1% bovine serum albumin and 0.001% sodium azide) and subjected to flow cytometric analysis.

### Flow cytometry

For flow cytometry, the following antibodies were used: PerCP/Cy5.5-conjugated mouse anti-pig CD4, PE-conjugated rat anti-human IL-10 (JES3-19 F1) and isotype controls PerCP/Cy5.5-conjugated mouse IgG2bκ, PE-conjugated rat IgG2ακ (BD Biosciences, San Jose, CA, USA), Alexa Fluor 647-conjugated anti-mouse/rat Foxp3 (FJK-16 s) (eBiosciences, San Diego, CA, USA), and isotype control Alexa Fluor 647-conjugated rat IgG2ακ (BioLegend, San Diego, CA, USA).

Lymphocytes isolated from the peripheral blood and intestine were incubated with 50 ng/mL of PMA (Sigma) and 5 mM calcium ionophore A23187 (Sigma) in the presence of Golgistop (BD Biosciences) in complete RPMI1640 medium for 4 h at 37 °C. After this in vitro stimulation, cells were washed with staining buffer (BD Biosciences) and surface CD4 was stained with PerCP/Cy5.5-conjugated anti-CD4 antibody for 30 min at 4 °C. Cells were washed with staining buffer, fixed in Cytofix/Cytoperm, permeabilized with Perm/Wash buffer (BD Biosciences), and intracellular staining was performed using PE-conjugated anti-IL-10 and Alexa Fluor 647-conjugated anti-Foxp3, or appropriate isotype control antibody for 30 min at 4 °C. Antibody-stained cells were analyzed on a FACScalibur™ flow cytometer (BD Biosciences) equipped with *FlowJo* software (Tree Star).

### Quantitative real-time PCR

Total RNA was extracted from the jejunal and ileal tissue samples using Trizol reagent (Invitrogen, Carlsbad, CA, USA), as previously described [15]. The integrity of RNA extracted from each sample was confirmed by agarose gel electrophoresis with ethidium bromide staining and visualization under UV light. A NanoDrop® ND-2000C spectrophotometer (Thermo Fisher Scientific, Wilmington, DE, USA) was used to determine the amount of RNA extracted and to verify its purity (OD_260_/OD_280_ absorption ratio of >1.9). A total of 1 μg of total RNA was then reverse transcribed into first-strand cDNA using the GoScript reverse transcription system (Promega, Madison, WI, USA). To inspect DNA contamination, a negative control (without enzyme) was included. Synthesized cDNA was stored at –20 °C prior to real-time PCR analysis.

An ABI 7500 real-time PCR System (Applied Biosystems, Foster City, CA, USA) was used for quantitative real-time PCR analyses. The sequences of the primers used are listed in Additional file 2. The cDNAs were amplified with SYBR® Premix DimerEraser™ (TakaRa Biotechnology Inc., Dalian, China) containing 2 μL of cDNA, 1.0 μM primers, 10 μL of 2× SYBR Premix DimerEraser, 0.4 μL of ROX (passive reference dye). A non-template control of nuclease-free water was included in each run. All reactions were conducted in triplicate. To quantify relative mRNA expression, the cycle threshold (C_T_) values of the target genes were normalized to the geometric mean of the C_T_ values of 3 selected housekeeping genes that were β-actin, glyceraldehyde-3-phosphate dehydrogenase (GAPDH) and hypoxanthine phosphoribosyl-transferase (HPRT), and the results are presented as fold change using the 2^−ΔΔCT^ method. The relative expression of target gene mRNA in each group was calculated using the following equations: ΔC_T_ = C_T target gene_ − C_T geometric mean of housekeeping genes_, and ΔΔC_T_ = ΔC_T treated group_ − ΔC_T control group_.

### Immunhistochemistry

Jejunal tissue samples fixed with 4% paraformaldehyde were embedded in paraffin, cut into 3-μm sections, and collected on silanized slides. After deparaffinization and hydration using xylenes, the slides were subjected to microwave for antigen retrieval and then cooled to room temperature. Endogenous peroxidase activity was quenched with 3% H_2_O_2_ in methanol for 20 min at room temperature, after which slides were incubated with goat serum of the species from which the secondary antibody was produced for 30 min. Subsequently, sections were incubated overnight at 4 °C in a humidified chamber with a 1:100 dilution of the rat anti-mouse Foxp3 polyclonal primary antibody (FJK-16 s, eBioscience, San Diego, CA, USA).

Foxp3 was then detected using a commercial immunoperoxidase staining kit (Vectastain Elite ABC Kit; Vector Laboratories, Burlingame, CA, USA). Briefly, the sections were incubated with a 1:200 dilution of biotinylated secondary antibody goat anti-rat IgG for 2 h at room temperature, followed by incubation with the avidin-biotin-peroxidase complex reagent for 1 h. Bound antibody conjugates were visualized using 3.3’-DAB (Zhongshan Golden Bridge Biotechnology Co., Beijing, China) as a chromogen, which produces a brown staining. Sections were counterstained with hematoxylin and mounted with glycerol gelatin. Negative controls prepared using the same procedure with the exception of replacing the primary antibody with PBS were included in each batch. The slides were visualized and photographed using an Olympus BX41 microscope (Olympus, Tokyo, Japan) equipped with a Canon EOS 550D camera head (Canon, Tokyo, Japan).

### Statistical analysis

The SAS statistical software package, version 9.3 (SAS Institute Inc., Cary, NC) was used for statistical analyses. Data were analyzed using the software’s PROC MIXED procedure. Besides, statistical evaluation of the incidence of diarrhea was carried out using Pearson’s chi-squared test. For analysis of non-normally distributed and repeated-measure data, the non-parametric Friedman’s test using SAS procedure FREQ was performed to compare diarrhea scores between treatments. Moreover, the non-parametric Wilcoxon-Mann-Whitney *U*-test using the SAS procedure was performed to compare small intestine histological scores between treatments.

For data from blood sample, the statistical model included the fixed effects of treatment, litter, sex, sampling time, interactions between treatments and sampling time, as well as the random effect of individual pigs within a treatment. In addition, a first-order autoregressive covariance structure was applied to the model to account for the correlation between measures at different times within individual pigs. For data from intestinal tissue or colonic content samples, the statistical model included the fixed effects of treatment, litter, sex, intestinal section or colonic content, interactions between treatments and intestinal section or colonic content, as well as random effects associated with individual pigs within a treatment. Natural logarithm transformation was performed for the ELISA data of TNF-α, IL-17A, IL-10 and IgA to yield a normal distribution prior to analysis. Differences between least-square means were compared using Tukey’s test. A *P*-value of <0.05 was considered statistically significant.

## Results

### Clinical status

Before F4^+^ ETEC/VTEC/EPEC challenge, all pigs had a normal rectal temperature: 39.3 ± 0.32 °C. At 48 h after challenge, an increase in rectal temperature to 40.2 ± 0.31 °C was observed in F4^+^ ETEC/VTEC/EPEC-challenged pigs compared with control (CONT) pigs (*P* = 0.022). The rectal temperature of HDBE pigs was also >40.0 °C at 72 h after F4^+^ ETEC/VTEC/EPEC challenge. The rectal temperature of both ETEC and HDBE pigs remained higher than that of CONT pigs until 144 h after challenge (*P* = 0.041 and *P* = 0.002, respectively), but this was not observed in LDBE pigs.

The effects of oral administration of a select mixture of BLS-mix on the incidence and duration of diarrhea in newly weaned pigs before and after F4^+^ ETEC/VTEC/EPEC challenge are shown in Additional file 3. During week 1 (prior to the F4^+^ ETEC/VTEC/EPEC challenge), 2 pigs in the CONT group, and 1 pig each in the ETEC and HDBE groups exhibited naturally acquired severe diarrhea lasting for 1 day; however, only 1 pig in the LDBE group had mild diarrhea by day 4 (Additional file 4A and Additional file 3). During week 2, only 1 pig in the ETEC group and 2 pigs in the HDBE group exhibited mild diarrhea lasting for 1 day following F4^+^ ETEC/VTEC/EPEC challenge (Additional file 4B and Additional file 3). The diarrhea score distributions in week 1 and week 2 for the 4 groups were unaffected by the challenge. When presented as pig-days with diarrhea, no difference in the incidence of diarrhea was found among the 4 groups.

In the first 2 weeks following the challenge, average daily weight gain and feed intake were lower in ETEC pigs compared with CONT pigs (*P* = 0.011 and *P* = 0.018, respectively), but there were no differences in these parameters in the LDBE or HDBE groups compared with CONT pigs (Additional file 5).

### Virulence factors produced by the inoculated F4^+^ ETEC/VTEC/EPEC strain

The presence of the selected virulence factors STb, LT, *stx2e*, as well as the LEE-encoded virulence factors *eae*, *Tir*, *escV* and *espA* were detected from the inoculated F4^+^ ETEC/VTEC/EPEC strain used in this study, whereas the other virulence factors STa, STh, STp, *cnf1*, *cnf2* were absent from this strain.

### Adhesion of F4^+^ ETEC/VTEC/EPEC to ileal mucosa

Immunofluorescence staining showed adhesion of F4^+^ ETEC/VTEC/EPEC strain to the ileal mucosa of pigs 1 week after challenge. The typical features associated with an attachment rating were as follows: 0 (Additional file 6A), no observed attachment of F4^+^ ETEC/VTEC/EPEC to the ileal mucosa; 1 (Additional file 6B); 2 (Additional file 6C); 3 (Additional file 6D); and 4 (Additional file 6E), F4^+^ ETEC/VTEC/EPEC were adhered to the crypt and entire villus.

### Effect of oral administration of BLS-mix on ameliorating F4^+^ ETEC/VTEC/EPEC- induced intestinal inflammation

**Figure 1:**
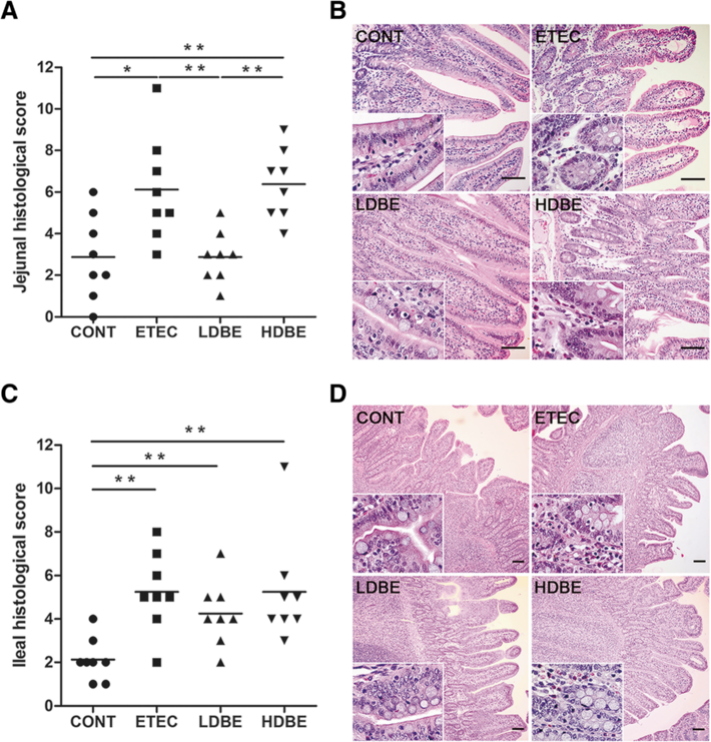
**Effect of oral administration of BLS-mix on F4** ^**+**^ **ETEC/VTEC/EPEC- associated enteritis in newly weaned F4ab/acR** ^**−**^ **pigs.** (**A**) Jejunal histological scores. (**B**) Representative photomicrographs of hematoxylin- and eosin-stained jejunal sections. Scale bars, 100 μm. (**C**) Ileal histological scores. (**D**) Representative photomicrographs of hematoxylin- and eosin-stained ileal sections. Scale bars, 100 μm. The F4 ab/acR^−^ pigs received sterile physiological saline orally (CONT), sterile physiological saline orally followed by F4^+^ ETEC/VTEC/EPEC (1.0 × 10^9^ CFU/mL, 10 mL, *per os* (p.o.)) challenge (ETEC), were pretreated with a low dose of BLS-mix (3.9 × 10^7^ CFU/mL, 10 mL once daily, p.o.) for 1 week followed by F4^+^ ETEC/VTEC/EPEC challenge (LDBE), or were pretreated with a high dose of BLS-mix (7.8 × 10^7^ CFU/mL, 10 mL once daily, p.o.) for 1 week followed by F4^+^ ETEC/VTEC/EPEC challenge (HDBE). *n* = 8 per group. *P < 0.05, **P < 0.01; non-parametric Wilcoxon-Mann-Whitney *U*-test.

On day 15 (1 week after F4^+^ ETEC/VTEC/EPEC challenge), histological analysis of the jejunum of ETEC and HDBE pigs showed increased intestinal inflammation compared with the jejunum of CONT pigs (*P* = 0.020 and *P* = 0.007, respectively), whereas lower histological scores were obtained for the jejunum of LDBE pigs compared with ETEC and HDBE pigs (*P* = 0.007 and *P* = 0.002, respectively; Figure 1A and B). Multiple foci of epithelial lesions were observed in the jejunum of ETEC and HDBE pigs. Central lacteal expansion, substantial leukocyte infiltration, and submucosa edema were also found in the jejunum of ETEC and HDBE pigs but not LDBE pigs.

Relative to CONT pigs, more severe intestinal inflammation was observed in the ileum of ETEC, LDBE, and HDBE pigs (*P* = 0.003, *P* = 0.008, *P* = 0.002, respectively; Figure 1C and D).

### Serum concentrations of TNF-α, IL-17A, IL-10 and IgA

**Figure 2:**
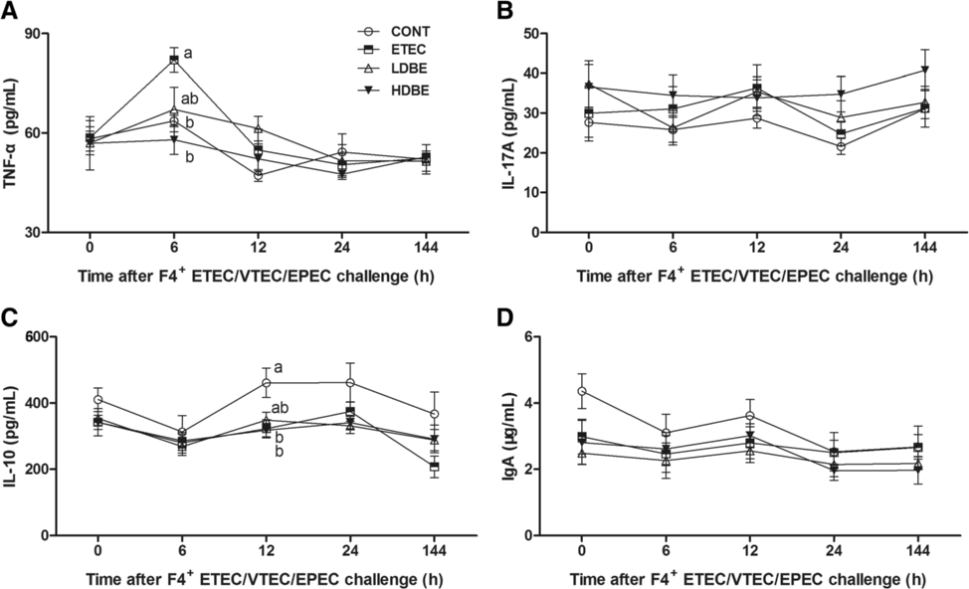
**Concentrations of TNF-α, IL-17A, IL-10, and IgA in the serum.** Blood samples were collected from the indicated pigs at 0, 6, 12, 24, and 144 h after F4^+^ ETEC/VTEC/EPEC challenge. The serum concentrations of (**A**) TNF-α, (**B**) IL-17A, (**C**) IL-10, and (**D**) IgA were determined by ELISA. Data are presented as the mean ± SEM for each time point (*n* = 8 per group). Mean values at the same time point without a common superscript (^a, b^) differ significantly (*P* < 0.05); Tukey’s test.

At 6 h after F4^+^ ETEC/VTEC/EPEC challenge, the serum concentration of TNF-α was elevated (*P* = 0.035) in ETEC pigs but not in either LDBE or HDBE pigs compared with CONT pigs (Figure 2A). Notably, at 12 h after challenge, the serum concentration of IL-10 was lower in ETEC and HDBE pigs than in CONT pigs (*P* = 0.022 and *P* = 0.018, respectively; Figure 2C). No differences in the serum concentrations of IL-17A or IgA were observed among the 4 groups (Figure 2B and D).

### Oral administration of BLS-mix increases the percentage of peripheral blood Foxp3^−^IL-10^+^ Treg cells

**Figure 3:**
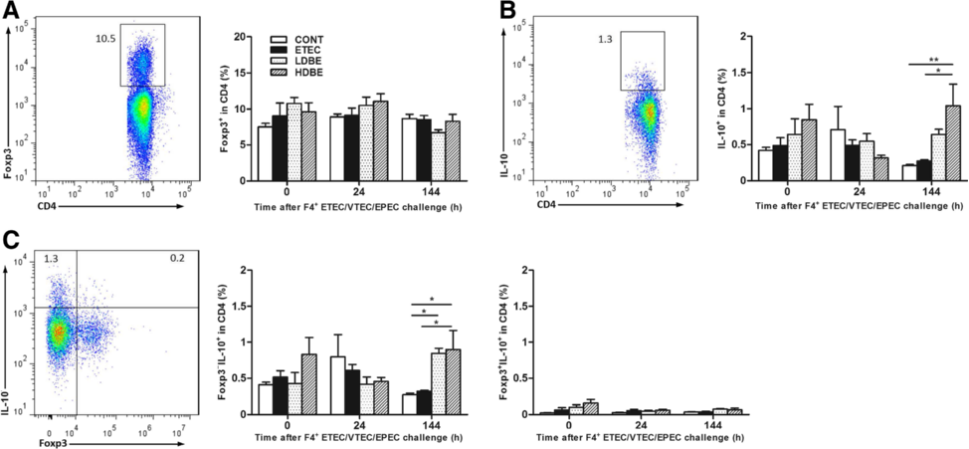
**Representative flow cytometry dot plots and peripheral blood Treg-cell population.** Peripheral blood samples were collected from the indicated pigs at 0, 24, and 144 h after F4^+^ ETEC/VTEC/EPEC challenge. Left, representative flow cytometry dot plots. Right, flow cytometry was used to determine the percentages of (**A**) Foxp3^+^, (**B**) IL-10^+^, (**C**) Foxp3^−^IL10^+^ and Foxp3^+^IL10^+^ Treg cells within the peripheral blood CD4^+^ T-cell population in the indicated pigs. Data are presented as the mean ± SEM for each time point (*n* = 8 per group). *P < 0.05; **P < 0.01; Tukey’s test.

Although the percentage of Foxp3^+^ Treg cells within the CD4^+^ T-cell population in the peripheral blood of ETEC, LDBE, and HDBE pigs was unaltered relative to CONT pigs at 0, 24, and 144 h after F4^+^ ETEC/VTEC/EPEC challenge, the percentage of CD4^+^IL-10^+^ T cells in the peripheral blood of HDBE pigs but not of LDBE pigs was higher at 144 h after challenge compared with that of either CONT or ETEC pigs (*P* = 0.007 and *P* = 0.019, respectively; Figure 3A and B). Both CD4^+^Foxp3^+^ and CD4^+^Foxp3^−^ T cells expressed IL-10 (Figure 3C). At 144 h after challenge, the percentage of peripheral blood Foxp3^−^IL-10^+^ Treg cells in LDBE and HDBE pigs was higher than that of CONT pigs (*P* = 0.029 and *P* = 0.015, respectively), and the percentage of Foxp3^−^IL-10^+^ Treg cells was higher in HDBE pigs than in ETEC pigs (*P* = 0.035). However, the percentage of Foxp3^+^IL-10^+^ Treg cells among CD4^+^ T cells in the peripheral blood of ETEC, LDBE, and HDBE pigs was no different at 0, 24, and 144 h after F4^+^ ETEC/VTEC/EPEC challenge compared with that of CONT pigs.

### The intestinal cytokine profile is altered in F4^+^ ETEC/VTEC/EPEC-challenged pigs pretreated with BLS-mix

**Figure 4:**
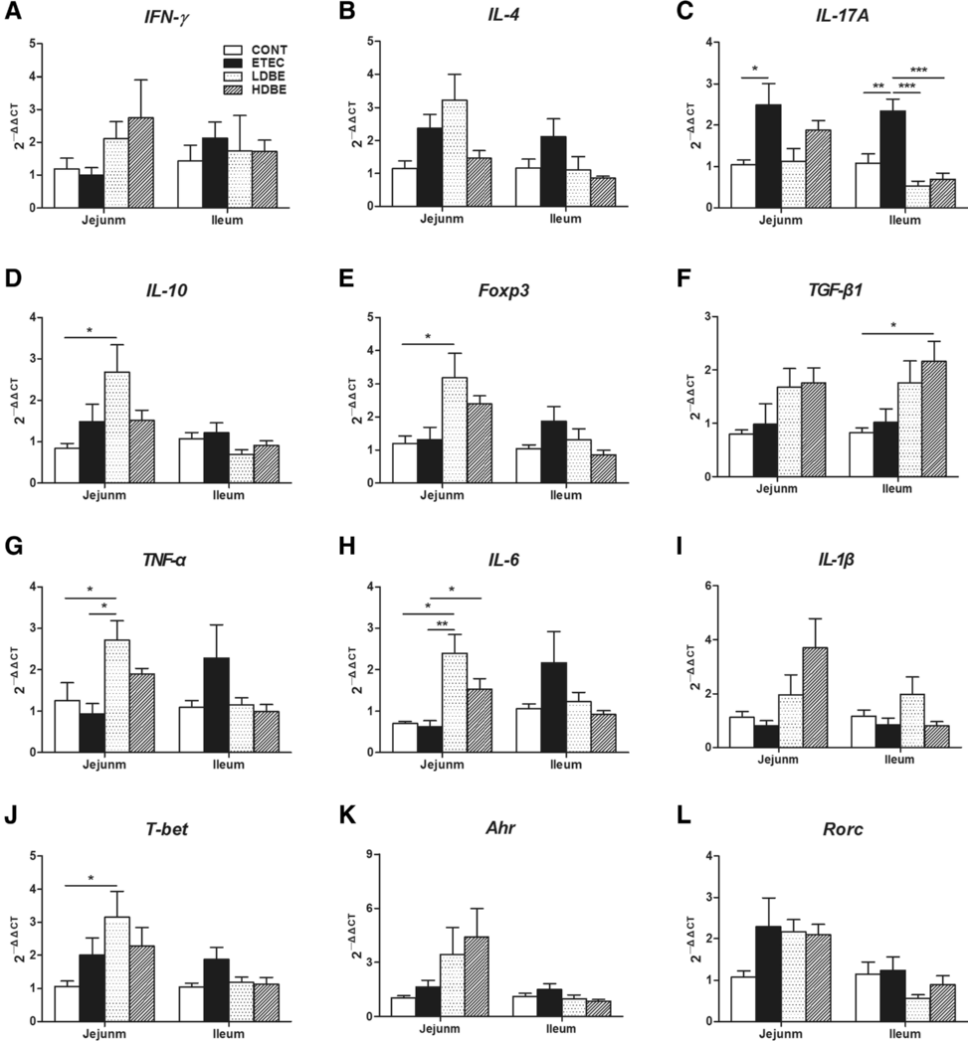
**Consumption of BLS-mix alters the cytokine profile in the small intestine after exposure to F4** ^**+**^ **ETEC/VTEC/EPEC.** The expression of mRNAs for genes encoding (**A**) IFN-γ, (**B**) IL-4, (**C**) IL-17A, (**D**) IL-10, (**E**) Foxp3, (**F**) TGF-β1, (**G**) TNF-α, (**H**) IL-6, (**I**) IL-1β, (**J**) T-bet, (**K**) Ahr, and (**L**) Rorc in both jejunal and ileal tissues collected from the indicated pigs 1 week after F4^+^ ETEC/VTEC/EPEC challenge was analyzed using quantitative real-time PCR. Data are presented as the mean ± SEM for each tissue (*n* = 8 per group). *P < 0.05; **P < 0.01; ***P < 0.001; Tukey’s test.

Following F4^+^ ETEC/VTEC/EPEC challenge, no differences were found in the expression of mRNAs for genes encoding IFN-γ or IL-4 in the intestinal tissues among the 4 groups (Figure 4A and B); however, the expression of *IL-17A* mRNA was upregulated (*P* = 0.031 and *P* = 0.004, respectively) in both the jejunal and ileal tissues of ETEC pigs but not of pigs pretreated with BLS-mix compared with that of CONT pigs (Figure 4C). The expression of jejunal *IL-10* mRNA was higher in LDBE pigs than in CONT pigs (*P* = 0.020; Figure 4D). Jejunal *Foxp3* mRNA expression in LDBE pigs was elevated compared with CONT pigs (*P* = 0.039; Figure 4E). Notably, *TGF-β1* mRNA expression was upregulated in the ileal tissues of HDBE pigs but not of LDBE pigs compared with that of CONT pigs (*P* = 0.027; Figure 4F).

In addition, an increase in jejunal *TNF-α* mRNA expression was observed in LDBE pigs, but not in HDBE pigs, compared with CONT and ETEC pigs (*P* = 0.034 and *P* = 0.010, respectively; Figure 4G). Unexpectedly, *IL-6* mRNA expression in the jejunal tissues of LDBE pigs was upregulated compared with that of either CONT or ETEC pigs (*P* = 0.021 and *P* = 0.003, respectively), and it was also higher in the jejunal tissues of HDBE pigs than in that of ETEC pigs (*P* = 0.027; Figure 4H). Expression of mRNA for the transcription factor gene *T-bet* was higher in the jejunal tissues of LDBE pigs than in that of CONT pigs (*P* = 0.039; Figure 4J). No changes were observed in the expression of mRNA for *IL-1β*, the transcription factor retinoic acid receptor − related orphan receptor-γt (*Rorc*), or aryl hydrocarbon receptor (*Ahr*) in any of the intestinal tissues analyzed in this study (Figure 4I, K and L).

### Oral feeding BLS-mix increases the proportion of Foxp3^−^IL-10^+^ but not that of Foxp3^+^IL-10^+^ Treg cells in the small intestine

**Figure 5:**
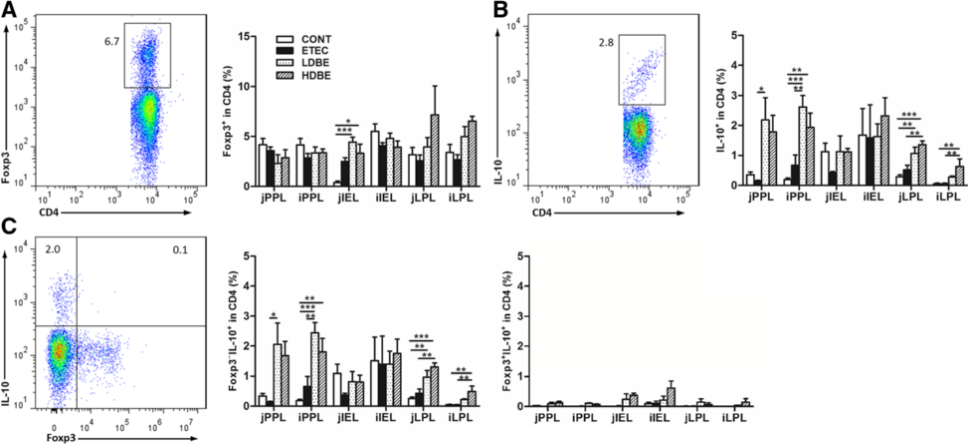
**BLS-mix increases the percentage of Foxp3** ^−^ **IL-10** ^**+**^ **but not Foxp3** ^**+**^ **IL-10** ^**+**^ **Treg cells in the small intestine.** Peyer’s patch lymphocytes (PPLs), intraepithelial lymphocytes (IELs), and lamina propria lymphocytes (LPLs) were isolated from both jejunal and ileal tissues collected from the indicated pigs 1 week after F4^+^ ETEC/VTEC/EPEC challenge. Left, representative flow cytometry dot plots. Right, flow cytometry was used to determine the percentage of (**A**) Foxp3^+^, (**B**) IL-10^+^, (**C**) Foxp3^−^IL10^+^ and Foxp3^+^IL10^+^ Treg cells within the CD4^+^ T-cell population in the small intestine. Data are presented as the mean ± SEM for each tissue (*n* = 8 per group). *P < 0.05; **P < 0.01; ***P < 0.001; Tukey’s test.

We next assessed changes in the populations of the 2 major regulatory T-cell types in the intestinal compartments, including the Peyer’s patches, the intraepithelial layer, and the lamina propria of the jejunum and ileum of pigs subjected to different treatments. There were no differences observed among the 4 groups with respect to the percentage of CD4^+^Foxp3^+^ Treg cells within PPLs or LPLs from either the jejunum or ileum (Figure 5A). However, the percentage of CD4^+^Foxp3^+^ Treg cells among jejunal intraepithelial lymphocytes (jIELs) was higher in LDBE and HDBE pigs than in CONT pigs (*P* < 0.001 and *P* = 0.015, respectively).

The percentage of CD4^+^IL10^+^ T cells among the jPPLs was higher in LDBE pigs than in ETEC pigs (*P* = 0.036; Figure 5B). Both LDBE and HDBE pigs had a higher percentage of CD4^+^IL10^+^ T cells among ileal PPLs (iPPLs) compared with CONT pigs (*P* < 0.001 and *P* = 0.008, respectively), and the percentage of ileal PPL CD4^+^IL10^+^ T cells in LDBE pigs but not in HDBE pigs was higher than that in ETEC pigs (*P* = 0.004). The percentage of jejunal LPL CD4^+^IL10^+^ T cells in both LDBE and HDBE pigs was higher than that in CONT pigs (*P* = 0.007 and *P* < 0.001, respectively), and it was higher in HDBE pigs than in ETEC pigs (*P* = 0.003). Notably, the percentage of ileal LPL CD4^+^IL10^+^ T cells was higher in HDBE pigs than in CONT or ETEC pigs (*P* = 0.008 and *P* = 0.006). There were no differences among the 4 groups with respect to the percentage of jejunal or ileal IEL CD4^+^IL10^+^ T cells.

Interestingly, the changes in the percentage of Foxp3^−^IL10^+^ Treg cells in the different intestinal compartments examined in this study mirrored the changes in the percentage of CD4^+^IL10^+^ T cells (Figure 5C). In contrast, no changes in the percentage of Foxp3^+^IL10^+^ Treg cells were observed in any of the intestinal tissues analyzed.

### Immunohistochemical localization of Foxp3^+^ Treg cells in the small intestine

**Figure 6:**
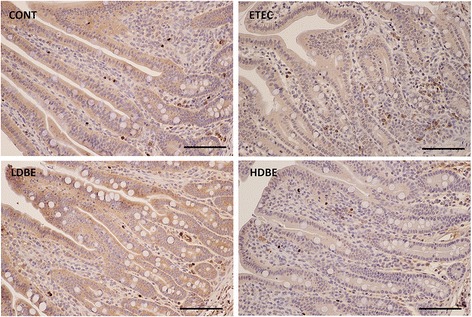
**Immunohistochemical localization of Foxp3 in the jejunum.** Representative photomicrographs of DAB-stained jejunal tissues collected from the indicated pigs 1 week after F4^+^ ETEC/VTEC/EPEC challenge. Foxp3^+^ Treg cells were primarily localized in the lamina propria and scattered in the epithelium. Scale bars, 100 μm.

Immunohistochemistry analysis was used to localize Foxp3^+^ Treg cells in the small intestine of pigs subjected to different treatments. Analyses of jejunum sections from all pigs revealed that Foxp3^+^ Treg cells were primarily localized in the lamina propria and scattered in the epithelium (Figure 6).

## Discussion

The outcome of experimental intestinal infections with *E. coli* is dependent on several factors. In the present study, the inoculated *E. coli* strain serotype/virotype profiles were O149:F4:LT:STb:Stx2e:eae, and we therefore named this uncommon strain as F4^+^ ETEC/VTEC/EPEC hybrid. In pigs, some strains (e.g. serogroup O149 isolates) of ETEC that cause postweaning diarrhea possess additional genes that encode Stx2e [1]. The incidence of diarrhea in newly weaned F4 ab/acR^−^ pigs was unchanged following F4^+^ ETEC/VTEC/EPEC challenge. However, oral inoculation of those pigs with F4^+^ ETEC/VTEC/EPEC resulted in enteritis in the small intestine and excessive systemic inflammatory responses including increased production of the proinflammatory cytokine TNF-α. We detected a transient increase in the serum TNF-α concentration in F4 ab/acR^−^ pigs after inoculation with F4^+^ ETEC/VTEC/EPEC, but this increase did not extend beyond 12 h post-challenge. Newly weaned F4 ab/acR^−^ pigs challenged with F4^+^ ETEC/VTEC/EPEC were particularly susceptible, and the sequence of clinical symptoms was generally fever, anorexia, depression, and weight loss. It is possible that the invading F4^+^ ETEC/VTEC/EPEC secrete heat-labile, heat-stable enterotoxins, Stx2e and the bacterial outer membrane protein intimin (*eae* gene) and release LPS, which in turn lead to intestinal inflammation and/or fever. A recent study showed that the toxin STb secreted by ETEC impaired intestinal epithelial barrier function by altering tight junction proteins [34]. Formation of attaching and effacing lesions on intestinal cells is associated with the presence of intimin secreted by EPEC/ETEC [35]. EspA is the main constituent of a filamentous structure and may act as a channel to deliver effector proteins into the host cell. Intimate attachment could be due to insertion of the bacterial protein Tir into the host epithelial cell membrane via the type III secretion system [36]. The VTEC strains produce Shiga-like toxin Stx2e cause edema disease in weaned piglets [37]. Besides, Stx2 induces autophagic cell death, also Stx2 treatment increases expression of IL-8 and TNF-α at both the protein and mRNA levels in human colonic epithelial cells [38,39]. Moreover, it has been identified STEC-, EPEC-, or/and ETEC-associated virulence genes coexisting in *E. coli* stains isolated from humans or animals [3,5]. These data therefore indicate that the polymorphism in MUC4 was not suitable to select all resistant pigs. It has been suggested that there is at least one other receptor for F4ab/ac *Escherichia coli* [40]. Thus, not only *MUC4* polymorphisms but also expression of other receptors should be included in any screening assay for F4ab/ac receptor − negative pigs [8].

Alternatively, specific antibodies against noninvasive *E. coli* could be employed at the intestinal mucosal surface to prevent ETEC attachment, colonization, and subsequent secretion of toxin. Polymeric and secretory IgA is best suited for mucosal surfaces; however, we found that IgA was present in the serum at concentrations <5 μg/mL, and that the concentration was unchanged after F4^+^ ETEC/VTEC/EPEC challenge in F4 ab/acR^−^ pigs, whether or not they were pretreated with BLS-mix. Consistent with our results, a previous study showed that F4R^−^ pigs remain F4-seronegative even after oral infection with F4^+^ ETEC or immunization with F4 fimbriae [41]. A recent study found that orally fed seeds producing designer IgAs protect newly weaned piglets against F4^+^ ETEC infection [42]. *Bacillus subtilis* could stimulate the mice GALT together with the production of specific sIgA responses [43]. Therefore, the effects of probiotic mixtures on porcine intestinal mucosal IgA responses should be examined in more detail in future studies.

Unexpectedly, we found that BLS-mix administration had no effect on the generation of peripheral blood CD4^+^Foxp3^+^ Treg cells in F4 ab/acR^−^ pigs, but a high dose of BLS-mix did result in an increase in the peripheral blood CD4^+^IL-10^+^ T-cell population. Furthermore, an increase in the proportion of Foxp3^−^IL-10^+^ T cells but not of Foxp3^+^IL-10^+^ T cells was found in pigs pre-treated by either a low or high dose of BLS-mix. In pigs, IL-10 is produced primarily by the CD4^+^CD25^dim^ cell subset, which contains a lower frequency of Foxp3^+^ T cells than does the CD4^+^CD25^high^ cell subset [26]. Human peripheral blood CD4^+^ T cells include a Foxp3^+^ T-cell subpopulation that does not exhibit suppressive activity but does produce proinflammatory cytokines upon activation [21]. Consistent with our previous study, here we found that the serum concentrations of IL-17 and IL-10 did not change after F4^+^ ETEC/VTEC/EPEC challenge in newly weaned piglets, although the pigs used in the previous study were not quantitatively screened for *MUC4* polymorphisms to determine the F4ab/ac receptor status [16]. It appears from the histological scores that only low dose of BLS-mix ameliorates enteritis in the jejunum but not in the ileum, whereas the high dose of BLS-mix makes it worse in both areas of the intestine. Our data indicate that the sheer presence of Foxp3^−^IL-10^+^ Treg cells cannot account for the protection of newly weaned F4ab/acR^−^ pigs from F4^+^ ETEC/VTEC/EPEC infection, and that excessive generation of CD4^+^IL-10^+^ T cells following consumption of BLS-mix during episodes of intestinal inflammation that is caused by enteric pathogens might prohibit clearance of the pathogen.

The results of gene expression profile analyses revealed that F4^+^ ETEC/VTEC/EPEC challenge in the absence of BLS-mix pretreatment had no effect on the expression of *IFN-γ*, *IL-1β*, or *IL-4* mRNAs in the small intestine but did result in increased expression of *IL-17A* mRNA in the jejunum and ileum. Moreover, increased expression of those mRNAs was attenuated by pretreatment with either a low or high dose of BLS-mix. Consistent with our results, a recent study showed that *IL-17* mRNA expression is upregulated in the intestine of pigs after infection with F4^+^ ETEC [44]. The most likely explanation for the results obtained in the present study is either that the presence of the heat-stable enterotoxin STb or the Shiga toxin Stx2 was sufficient to induce an IL-17A response [44,45] or that the Th17 repertoire was shaped by the specific microbiota to limit responses to luminal antigens [46]. In addition, administration of the probiotic *Lactobacillus casei* was shown to attenuate antigen-induced secretion of IL-17 by CD4^+^ T cells developed from Peyer’s patch cells [47].

Interestingly, we found that oral administration of a low dose of BLS-mix resulted in increased expression of mRNAs for the transcription factors *Foxp3* and *T-bet* associated with alternative CD4 T-cell programs in the jejunum of F4 ab/acR^−^ pigs after F4^+^ ETEC/VTEC/EPEC challenge. Immunohistochemical analyses of jejunal tissues revealed that Foxp3^+^ Treg cells were primarily localized in the lamina propria and scattered in the epithelium. Correspondingly, oral administration of BLS-mix was sufficient to increase the percentage of CD4^+^Foxp3^+^ cells among jejunal intraepithelial lymphocytes in newly weaned F4 ab/acR^−^ pigs following F4^+^ ETEC/VTEC/EPEC challenge. *Clostridium* species have been shown to induce an increase in the number of Foxp3^+^ Treg cells in the colon through TGF-β induction of epithelial cells [48]. Taken together, our results thus suggest that oral administration of BLS-mix could induce an increase in the number of CD4^+^Foxp3^+^ Treg cells among intraepithelial lymphocytes in the small intestine, as these cells have cytolytic and immunoregulatory capabilities and can be recruited quickly to maintain epithelial integrity and protect the host from infectious agents.

Here, we show that consumption of a select probiotic mixture, leads to increased expression of *TNF-α*, *IL-6*, *TGF-β1*, and *IL-10* mRNAs in the small intestine of F4 ab/acR^−^ pigs following F4^+^ ETEC/VTEC/EPEC challenge. It has been shown that IL-6 inhibits TGF-β − induced generation of Foxp3^+^ Treg cells [49]. Neither IL-6 nor IL-23 alone generates Th17 cells; however, in combination with IL-1β, these cytokines effectively induce IL-17 production [50]. In the present study, probiotic mixture inoculation of pigs has no effect on the expression of either *IL-1β* mRNA or mRNAs for the transcription factor *Rorc* which directs Th17-cell responses or another transcription factor *Ahr* in the small intestine of pigs following F4^+^ ETEC/VTEC/EPEC challenge. On the other hand, a recent study showed that IL-6 induces the generation of Tr1 cells from naïve CD4 T cells and suppresses LPS-induced inflammatory responses in an IL-10 − dependent fashion [51]. Our previous study demonstrated that following F4^+^ ETEC/VTEC/EPEC challenge, expression of *TLR2*, *TLR9*, and *NOD1* mRNA is upregulated in the intestines of pigs pretreated with a low, but not a high, dose of *L. rhamnosus* [15]. It has been postulated that TLR9-mediated activation of IL-6 signaling (and perhaps IL-27 signaling, which can induce production of the IL-10 − promoting cytokine IL-21) could induce IL-10 production and promote Tr1-like cell responses [24]. Our findings suggest that IL-6 may mediate Tr1-like cell responses and induce IL-10 production in the small intestine.

Consistent with our observations, a previous study showed that most *Bacillus* probiotics are excreted following oral inoculation [52]. However, *Bacillus subtilis* spores have been found in the Peyer’s patches and mesenteric lymph nodes in mice [43]. Notably, we found that oral administration of BLS-mix led to an increase in the percentage of CD4^+^IL-10^+^ T cells both in the Peyer’s patches and in the lamina propria in the small intestine of F4 ab/acR^−^ pigs following F4^+^ ETEC/VTEC/EPEC challenge. Furthermore, our results show that the increased number of IL-10–producing cells within the CD4^+^ T-cell population is primarily attributable to an increase in the proportion of Foxp3^−^IL-10^+^ rather than Foxp3^+^IL-10^+^ Treg cells. Similarly, *Bifidobacterium breve* administration leads to an increase in the number of IL-10–producing cells within the Foxp3^−^ but not Foxp3^+^ colonic CD4^+^ T-cell population in mice [27]. While the low dose of BLS-mix induced Tr1 cells and ameliorated some pathophysiological changes caused by F4^+^ ETEC/VTEC/EPEC infection, the high dose of BLS-mix did not prevent pathophysiological changes in the jejunum despite the fact that the number of Foxp3^−^IL-10^+^ T cells was increased both in the blood as well as the intestinal mucosa. This discrepancy suggests that the induction of IL-10–producing Tr1 cells is not the mechanism by which BLS-mix suppresses F4^+^ ETEC/VTEC/EPEC infection. Further studies are needed to clarify the mechanisms of the effectors involved in regulating the inflammatory response.

In the present study, we show that oral inoculation of newly weaned F4 ab/acR^−^ pigs with F4^+^ ETEC/VTEC/EPEC results in enteritis and excessive systemic inflammatory responses, and that oral administration of either a low or high dose of BLS-mix leads to an increase in the proportion of Foxp3^−^IL-10^+^ but not of Foxp3^+^IL-10^+^ Treg cells both in the blood as well as the intestinal mucosa of F4 ab/acR^−^ pigs following F4^+^ ETEC/VTEC/EPEC challenge. Our data indicate that the induction of IL-10–producing Tr1 cells by BLS-mix cannot account for the protection of newly weaned F4ab/acR^−^ pigs from F4^+^ ETEC/VTEC/EPEC infection, and that excessive generation of CD4^+^IL-10^+^ T cells following consumption of BLS-mix during episodes of intestinal inflammation that is caused by enteric pathogens might prohibit clearance of the pathogen.

## Additional files

Additional file 1:
**Sequences of oligonucleotide primers used for real-time PCR, length of the respective PCR product and gene accession number.** The table shows the sequences of primers used for real-time PCR in this study. Additional file 2:
**Sequences of oligonucleotide primers used for quantitative real-time PCR, length of the respective PCR product and gene accession number.** The table shows the sequences of primers used for quantitative real-time PCR in this study. Additional file 3:
**Dose effects of oral administration of BLS-mix on the incidence and duration of diarrhea in newly weaned pigs before and after F4** ^**+**^ **ETEC/VTEC/EPEC challenge.** The table shows the incidence and duration of diarrhea in newly weaned pigs for 2 weeks. The diarrhea score distributions in week 1 and week 2 for pigs subjected to different treatments were unaffected by the challenge. No difference in the incidence of diarrhea was found among the 4 groups (*P* > 0.05). Additional file 4:
**Effects of BLS-mix on diarrhea scores of newly weaned F4ab/acR** ^**−**^ **pigs following F4** ^**+**^ **ETEC/VTEC/EPEC challenge.** The figures show the distribution of diarrhea scores in (A) week 1 (before F4^+^ ETEC/VTEC/EPEC challenge) and (B) week 2 (after F4^+^ ETEC/VTEC/EPEC challenge) for the indicated pigs. Data are presented as the frequency of each diarrhea score within pig days (n = 8 pigs per group); No difference between the indicated groups was observed (*P* > 0.05); non-parametric Friedman’s test. Additional file 5:
**Dose effect of oral administration of BLS-mix on the growth of newly weaned pigs before and after F4** ^**+**^ **ETEC/VTEC/EPEC challenge.** The table shows the growth of newly weaned pigs before and after F4^+^ ETEC/VTEC/EPEC challenge. In the first 2 weeks following the challenge, average daily weight gain and feed intake were lower in ETEC pigs compared with CONT pigs (*P* = 0.011 and *P* = 0.018, respectively), but there were no differences relative to the CONT pigs in these parameters in the LDBE or HDBE groups. Additional file 6:
**Immunofluorescence staining of F4** ^**+**^ **ETEC/VTEC/EPEC strain in the ileum.** The figures show representative photomicrographs of F4^+^ ETEC/VTEC/EPEC (red) adhesion to the ileal mucosa of pigs 1 week after F4^+^ ETEC/VTEC/EPEC challenge. The typical features associated with an attachment rating were as follows: 0 (A), no observed attachment of F4^+^ ETEC/VTEC/EPEC to the ileal mucosa; 1 (B); 2 (C); 3 (D); and 4 (E), F4^+^ ETEC/VTEC/EPEC were adhered to the crypt and entire villus. Scale bars, 50 μm.

## Abbreviations

Ahr: aryl hydrocarbon receptor
BLS-mix: *Bacillus licheniformis* and *Bacillus subtilis* mixture
F4^+^ ETEC: F4 (K88)-positive enterotoxigenic *Escherichia coli*
VTEC: verocytotoxigenic *Escherichia coli*
EPEC: enteropathogenic *Escherichia coli*
F4R^−^: F4 receptor − negative
IEL: intraepithelial lymphocyte
LPL: lamina propria lymphocyte
PPL: Peyer’s patch lymphocyte
Rorc: retinoic acid receptor–related orphan receptor-γt
Tr1: T regulatory type 1 cell
Treg: regulatory T cell
