# Supplementary material for: Oral administration of a select mixture of Bacillus probiotics generates Tr1 cells in weaned F4ab/acR− pigs challenged with an F4+ ETEC/VTEC/EPEC strain
Source: Vet Res. 2015 Sep 17;46(1):95. doi: 10.1186/s13567-015-0223-y (PMC4574530; doi:10.1186/s13567-015-0223-y)
Supplement: Additional file 1: — Sequences of oligonucleotide primers used for real-time PCR, length of the respective PCR product and gene accession number. The table shows the sequences of primers used for real-time PCR in this study. [file 13567_2015_223_MOESM1_ESM.doc]

**Additional file 1 Sequences of oligonucleotide primers used for real-time PCR, length of the respective PCR product and gene accession number.**

| **Gene** | **Primer** | | **Product** | | **Accession** |  |
| --- | --- | --- | --- | --- | --- | --- |
| **product*^a^*** | **Direction*****^b^*** | **Sequence (5'→3')** | **size (bp)** | | **number** | **References** |
| Sta | F | ATGTTGGCAATTTTTATTTCTGTA | | 183 | M25607.1 | [53] |
|  | R | ATTACAACAAAGTTCACAGCAGTA | |  |  |  |
| STh | F | CCTTTCGCTCAGGATGCTAAAC | | 128 | AY342058.1 | [32] |
|  | R | CAGTAATTGCTACTATTCATGCTTTCAG | |  |  |  |
| STp | F | CTTTCCCCTCTTTTAGTCAGTCAACT | | 137 | M58746.1 | [32] |
|  | R | GCAGTAAAATGTGTTGTTCATATTTTCTG | |  |  |  |
| STb | F | ATGTAAATACCTACAACGGGTGAT | | 360 | AY028790.1 | [53] |
|  | R | TATTTGGGCGCCAAAGCATGCTCC | |  |  |  |
| LT | F | TTCCCACCGGATCACCAA | | 62 | KF733767.1 | [32] |
|  | R | CAACCTTGTGGTGCATGATGA | |  |  |  |
| *Stx2e*^*^ | F | ATACGATGACGCCGGAAGAC | | 291 | U72191.1 |  |
|  | R | TCAGAAACGCTGCTGCTGTA | |  |  |  |
| *cnf1* | F | AAGATGGAGTTTCCTATGCAGGAG | | 498 | X70670.1 | [54] |
|  | R | CATTCAGAGTCCTGCCCTCATTATT | |  |  |  |
| *cnf2*^*^ | F | GCGGAAATTTGAGCGGTTGT | | 165 | U01097.1 |  |
|  | R | CGCGCGGCATTGGATTATTT | |  |  |  |
| *eae* | F | CCGATTCCTCTGGTGACGA | | 105 | AB647618.1 | [32] |
|  | R | CCACGGTTTATCAAACTGATAACG | |  |  |  |
| *Tir*^*^ | F | GTTGGCTTTGACACCGGAAC | | 379 | AF022236 |  |
|  | R | TACACCAGCACCAATTCCCC | |  |  |  |
| *escV* | F | ATTCTGGCTCTCTTCTTCTTTATGGCTG | | 544 | AF022236 | [5] |
|  | R | CGTCCCCTTTTACAAACTTCATCGC | |  |  |  |
| *espA* | F | TCAGAATCGCAGCCTGAAAA | | 60 | AF022236 | [33] |
|  | R | CGAAGGATGAGGTGGTTAAGCT | |  |  |  |

*^a^* ST, heat-stable enterotoxin; LT, heat-labile enterotoxin; *Stx*, Shiga-like toxin; *cnf*, cytotoxic necrotizing factor; *eae*, *E. coli* attaching and effacing gene; *Tir*, translocated intimin receptor; *esp*A, *E. coli*-secreted protein A.

*^b^* F, forward; R, reverse.

^*^ The oligonucleotide primers were designed in the present study.
