# Supplementary material for: Oral administration of a select mixture of Bacillus probiotics generates Tr1 cells in weaned F4ab/acR− pigs challenged with an F4+ ETEC/VTEC/EPEC strain
Source: Vet Res. 2015 Sep 17;46(1):95. doi: 10.1186/s13567-015-0223-y (PMC4574530; doi:10.1186/s13567-015-0223-y)
Supplement: Additional file 2: — Sequences of oligonucleotide primers used for quantitative real-time PCR, length of the respective PCR product and gene accession number. The table shows the sequences of primers used for quantitative real-time PCR in this study. [file 13567_2015_223_MOESM2_ESM.doc]

**Additional file 2 Sequences of oligonucleotide primers used for quantitative real-time PCR, length of the respective PCR product and gene accession number.**

| **Gene** | **Primer** | | **Product** |  |
| --- | --- | --- | --- | --- |
| **product*^a^*** | **Direction*****^b^*** | **Sequence (5'→3')** | **size (bp)** | **Accession number** |
| HPRT | F | GTGATAGATCCATTCCTATGACTGTAGA | 104 | U69731 |
|  | R | TGAGAGATCATCTCCACCAATTACTT |  |  |
| GAPDH | F | CCAGAACATCATCCCTGCTT | 229 | NM_001206359.1 |
|  | R | GTCCTCAGTGTAGCCCAGGA |  |  |
| β-actin | F | CTCTTCCAGCCCTCCTTCCT | 103 | XM_003357928.2 |
|  | R | GCGTAGAGGTCCTCCTGATGT |  |  |
| IFN-γ | F | TAAATGGTAGCTCTGGGAAACTGAA | 86 | NM_213948 |
|  | R | GATGGCTTTGCGCTGGA |  |  |
| IL-4 | F | ACACAAGTGCGACATCACCTTA | 132 | NM_214123 |
|  | R | GTTTCCTTCTCCGTCGTGTTCT |  |  |
| IL-17A | F | CGGCTGGAGAAAGTGATGGT | 140 | NM_001005729 |
|  | R | CAGAAATGGGGCTGGGTCT |  |  |
| IL-10 | F | CCTGACTGCCTCCCACTTTC | 94 | NM_214041 |
|  | R | GGGCTCCCTAGTTTCTCTTCCT |  |  |
| Foxp3 | F | GGTGCAGTCTCTGGAACAAC | 148 | NM_001128438 |
|  | R | GGTGCCAGTGGCTACAATAC |  |  |
| TGF-β1 | F | GAAGCGCATCGAGGCCATTC | 162 | NM_214015 |
|  | R | GGCTCCGGTTCGACACTTTC |  |  |
| TNF-α | F | GCCCACGTTGTAGCCAATGTCAAA | 99 | NM_214022 |
|  | R | GTTGTCTTTCAGCTTCACGCCGTT |  |  |
| IL-6 | F | GGGAAATGTCGAGGCTGTG | 91 | NM_214399 |
|  | R | AGGGGTGGTGGCTTTGTCT |  |  |
| IL-1β | F | GGCCGCCAAGATATAACTGA | 70 | NM_214055 |
|  | R | GGACCTCTGGGTATGGCTTTC |  |  |
| T-bet | F | TGGACCCAACTGTCAATTGCT | 76 | XM_003131530 |
|  | R | ACGGCTGGGAACGGGATA |  |  |
| Ahr | F | AGCTGCACTGGGCGTTAAA | 68 | XM_005667666.1 |
|  | R | GCCACTCGCTTCATCAATTCT |  |  |
| Rorc | F | GAAGTGGTGCTGGTCAGGAT | 140 | XM_003355171 |
|  | R | CGGGAGAAGTCAAAGATGGA |  |  |

*^a^* HPRT, hypoxanthine phosphoribosyl-transferase; GAPDH, glyceraldehyde-3-phosphate dehydrogenase; Foxp3, forkhead box P3; T-bet, T-box protein 21; Ahr, aryl hydrocarbon receptor; Rorc, retinoic-acid-receptor-related orphan receptor-γ.

*^b^* F, forward; R, reverse.
