# Supplementary material for: Oral administration of a select mixture of Bacillus probiotics generates Tr1 cells in weaned F4ab/acR− pigs challenged with an F4+ ETEC/VTEC/EPEC strain
Source: Vet Res. 2015 Sep 17;46(1):95. doi: 10.1186/s13567-015-0223-y (PMC4574530; doi:10.1186/s13567-015-0223-y)
Supplement: Additional file 3: — Dose effects of oral administration of BLS-mix on the incidence and duration of diarrhea in newly weaned pigs before and after F4 + ETEC/VTEC/EPEC challenge. The table shows the incidence and duration of diarrhea in newly weaned pigs for 2 weeks. The diarrhea score distributions in week 1 and week 2 for pigs subjected to different treatments were unaffected by the challenge. No difference in the incidence of diarrhea was found among the 4 groups (P > 0.05). [file 13567_2015_223_MOESM3_ESM.doc]

**Additional file 3 Dose effects of oral administration of BLS-mix on the incidence and duration of diarrhea in newly weaned pigs before and after F4^+^ ETEC/VTEC/EPEC challenge.**

| **Group*^a^*/item** | **Pigs** |  |  |  | **Pig days** |  |  |  |  |  |  |
| --- | --- | --- | --- | --- | --- | --- | --- | --- | --- | --- | --- |
|  | **At risk** | **With diarrhea** | |  | **At risk** | **With diarrhea** | |  | **Significance of difference** | | |
|  | **(n)** | **(n)** | **(%)** |  | **(n)** | **(n)** | **(%)** |  | **ETEC** | **LDBE** | **HDBE** |
| Week 1 |  |  |  |  |  |  |  |  |  |  |  |
| CONT | 8 | 2 | 25 |  | 56 | 2 | 3.6 |  | 0.164 | 0.078 | 0.234 |
| ETEC | 8 | 1 | 12.5 |  | 56 | 1 | 1.8 |  |  | 0.331 | 0.333 |
| LDBE | 8 | 1 | 12.5 |  | 56 | 1 | 1.8 |  |  |  | 0.499 |
| HDBE | 8 | 1 | 12.5 |  | 56 | 1 | 1.8 |  |  |  |  |
|  |  |  |  |  |  |  |  |  |  |  |  |
| Week 2 |  |  |  |  |  |  |  |  |  |  |  |
| CONT | 8 | 0 | 0 |  | 56 | 0 | 0 |  | 0.667 | 0.055 | 0.176 |
| ETEC | 8 | 1 | 12.5 |  | 56 | 1 | 1.8 |  |  | 0.095 | 0.603 |
| LDBE | 8 | 0 | 0 |  | 56 | 0 | 0 |  |  |  | 0.179 |
| HDBE | 8 | 2 | 25 |  | 56 | 2 | 3.6 |  |  |  |  |

*^a^* F4ab/acR^−^ piglets received sterile physiological saline orally (CONT), received sterile physiological saline orally followed by F4^+^ ETEC/VTEC/EPEC (1.0 × 10^9^ CFU/mL, 10 mL, p.o.) challenge (ETEC), were pretreated with a low dose of the mixture of *Bacillus licheniformis* and *Bacillus subtilis* (BLS-mix, 3.9 × 10^7^ CFU/mL, 10 mL once daily, p.o.) for 1 week followed by F4^+^ ETEC/VTEC/EPEC challenge (LDBE), or were pretreated with a high dose of BLS-mix (7. 8 × 10^7^ CFU/mL, 10 mL once daily, p.o.) for 1 week followed by F4^+^ ETEC/VTEC/EPEC challenge (HDBE).

*n* = 8 pigs per group; Pearson’s chi-squared test.
