# Supplementary figures and images for: Oral administration of a select mixture of Bacillus probiotics generates Tr1 cells in weaned F4ab/acR− pigs challenged with an F4+ ETEC/VTEC/EPEC strain
Source: Vet Res. 2015 Sep 17;46(1):95. doi: 10.1186/s13567-015-0223-y (PMC4574530; doi:10.1186/s13567-015-0223-y)

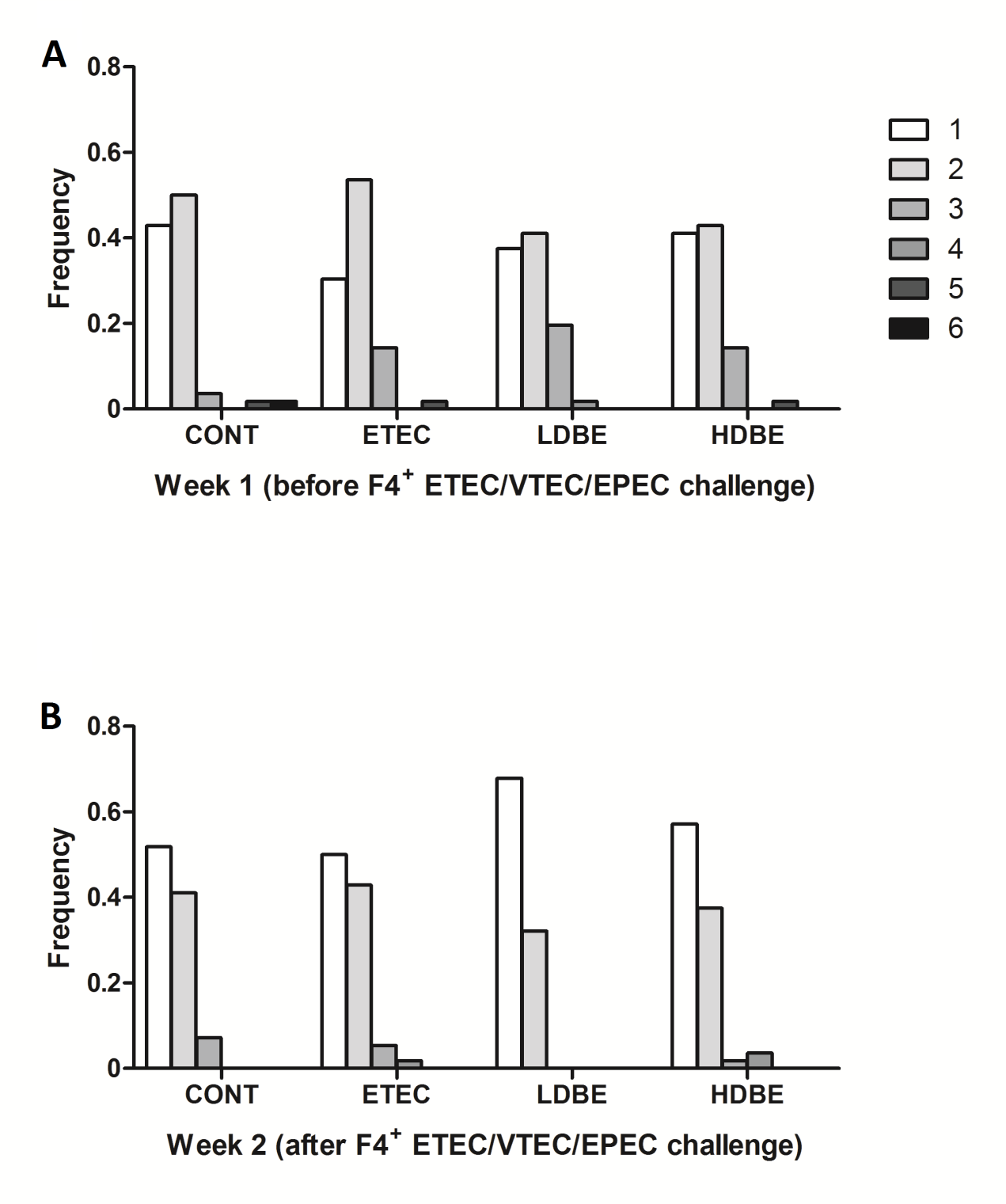

Supplement: Additional file 4: — Effects of BLS-mix on diarrhea scores of newly weaned F4ab/acR − pigs following F4 + ETEC/VTEC/EPEC challenge. The figures show the distribution of diarrhea scores in (A) week 1 (before F4+ ETEC/VTEC/EPEC challenge) and (B) week 2 (after F4+ ETEC/VTEC/EPEC challenge) for the indicated pigs. Data are presented as the frequency of each diarrhea score within pig days (n = 8 pigs per group); No difference between the indicated groups was observed (P > 0.05); non-parametric Friedman’s test. [file 13567_2015_223_MOESM4_ESM.doc]

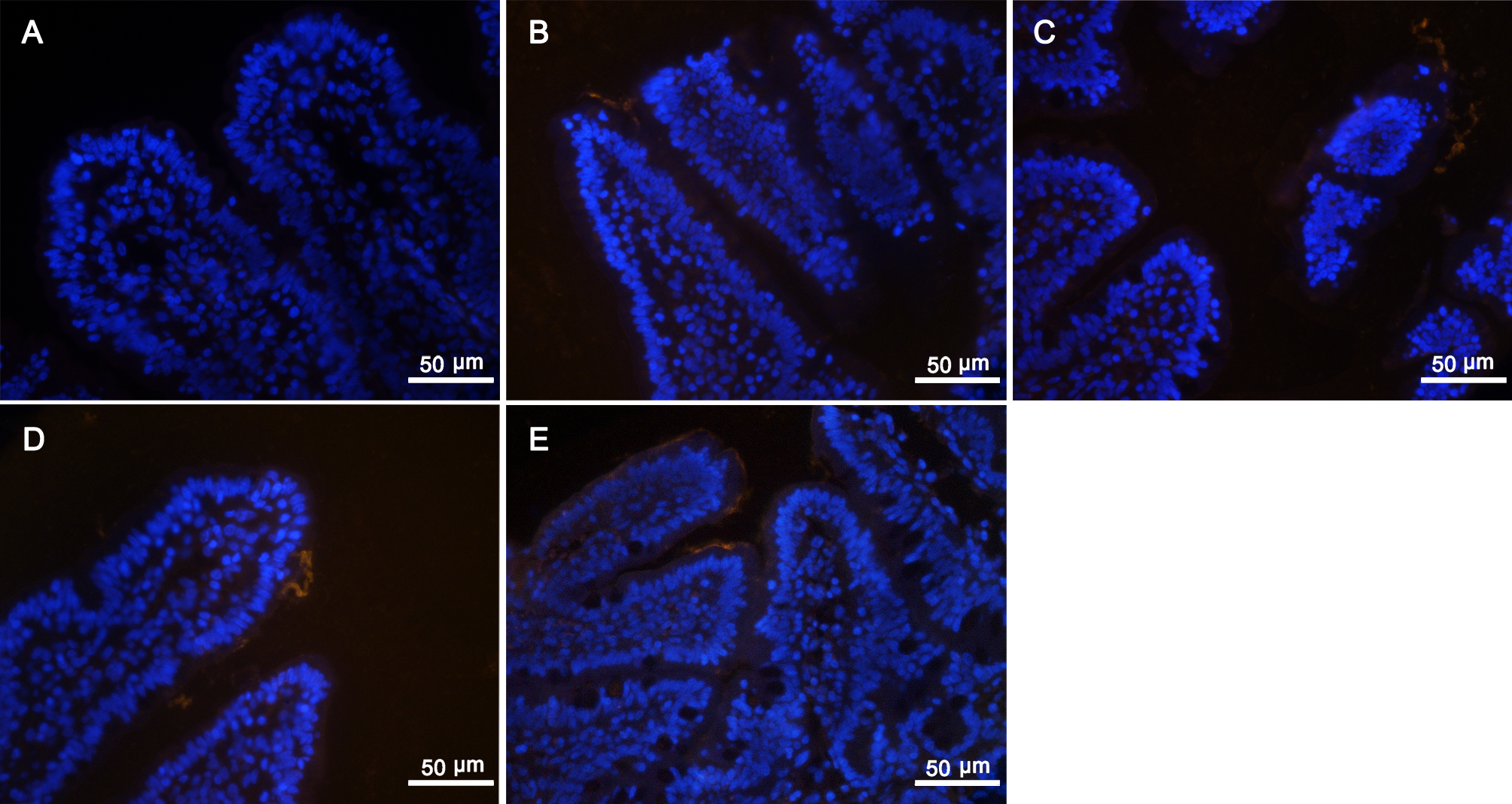

Supplement: Additional file 6: — Immunofluorescence staining of F4 + ETEC/VTEC/EPEC strain in the ileum. The figures show representative photomicrographs of F4+ ETEC/VTEC/EPEC (red) adhesion to the ileal mucosa of pigs 1 week after F4+ ETEC/VTEC/EPEC challenge. The typical features associated with an attachment rating were as follows: 0 (A), no observed attachment of F4+ ETEC/VTEC/EPEC to the ileal mucosa; 1 (B); 2 (C); 3 (D); and 4 (E), F4+ ETEC/VTEC/EPEC were adhered to the crypt and entire villus. Scale bars, 50 μm. [file 13567_2015_223_MOESM6_ESM.tiff]
