# Supplementary material for: Oral administration of a select mixture of Bacillus probiotics generates Tr1 cells in weaned F4ab/acR− pigs challenged with an F4+ ETEC/VTEC/EPEC strain
Source: Vet Res. 2015 Sep 17;46(1):95. doi: 10.1186/s13567-015-0223-y (PMC4574530; doi:10.1186/s13567-015-0223-y)
Supplement: Additional file 5: — Dose effect of oral administration of BLS-mix on the growth of newly weaned pigs before and after F4 + ETEC/VTEC/EPEC challenge. The table shows the growth of newly weaned pigs before and after F4+ ETEC/VTEC/EPEC challenge. In the first 2 weeks following the challenge, average daily weight gain and feed intake were lower in ETEC pigs compared with CONT pigs (P = 0.011 and P = 0.018, respectively), but there were no differences relative to the CONT pigs in these parameters in the LDBE or HDBE groups. [file 13567_2015_223_MOESM5_ESM.doc]

**Additional file 5 Dose effect of oral administration of BLS-mix on the growth of newly weaned pigs before and after F4+ ETEC/VTEC/EPEC challenge.**

| **Item^2^** | **Treatment^1^** | | | | **SEM** | ***P*-value** |
| --- | --- | --- | --- | --- | --- | --- |
|  | **CONT** | **ETEC** | **LDBE** | **HDBE** |  |  |
| Week 1 (days 0 to 8, pre-challenge) |  |  |  |  |  |  |
| ADG, g/d | 127 | 136 | 133 | 111 | 12 | 0.849 |
| ADFI, g/d | 159 | 146 | 145 | 147 | 12 | 0.916 |
| G:F, g/g | 0.80 | 0.93 | 0.92 | 0.76 | 0.08 | 0.941 |
| Week 2 (days 8 to 15, post-challenge) |  |  |  |  |  |  |
| ADG, g/d | 287^a^ | 106^b^ | 233^ab^ | 234^ab^ | 20 | 0.210 |
| ADFI, g/d | 310^a^ | 218^b^ | 318^a^ | 324^a^ | 11 | 0.115 |
| G:F, g/g | 0.93 | 0.49 | 0.73 | 0.72 | 0.06 | 0.673 |

^1^ F4ab/acR^−^ piglets received sterile physiological saline orally (CONT), received sterile physiological saline orally followed by F4^+^ ETEC/VTEC/EPEC (1.0 × 10^9^ CFU/mL, 10 mL, p.o.) challenge (ETEC), were pretreated with a low dose of the mixture of *Bacillus licheniformis and Bacillus subtilis* (BLS-mix, 3.9 × 10^7^ CFU/mL, 10 mL once daily, p.o.) for 1 week followed by F4^+^ ETEC/VTEC/EPEC challenge (LDBE), or were pretreated with a high dose of BLS-mix (7. 8 × 10^7^ CFU/mL, 10 mL once daily, p.o.) for 1 week followed by F4^+^ ETEC/VTEC/EPEC challenge (HDBE). *n* = 8 per group.

^a, b^ Within a row, least-square means without a common lowercase superscript differ (*P*< 0.05); Tukey’s test.

^2^ ADG, average daily gain; ADFI, average daily feed intake; G: F, gain to feed ratio.
